# Supplementary material for: Content-rich biological network constructed by mining PubMed abstracts
Source: BMC Bioinformatics. 2004 Oct 8;5:147. doi: 10.1186/1471-2105-5-147 (PMC528731; doi:10.1186/1471-2105-5-147)
Supplement: Additional File 2 — The original results of the above study (non-essential files are deleted to keep the file size under the limit set by BMC bioinformatics). [file 1471-2105-5-147-S2.bz2 › chilibotAdditionalFile2/dip10/482ID8702798E10572/html/IL8RA_CXCL5.html]

 


 **IL8RA** and **CXCL5** 
  
Found 14 abstracts in PubMed, retrieved 10.  
 

 What does Google say? 
 PDF only 
| .edu only 

---

**Interactive relationship** (e.g. stimulation, inhibition, etc)

**Stimulatory relationship**- GCP 2 desensitized the calcium increase induced by IL 8 in both CXCR1  [ **IL8RA** ]  transfected and CXCR2 transfected cells, but ENA 78  [ **CXCL5** ]  only affected the IL 8 induced calcium response in CXCR2 transfectants.  Ref: 9692902 Eur J Biochem, 1998
**Neutral relationship**- IL 8 activates neutrophils by binding to two distinct seven transmembrane 7 TMR G protein coupled receptors CXCR1  [ **IL8RA** ]  IL 8RA and CXCR2 IL 8RB, while GROalpha, GRObeta, GROgamma, and ENA 78  [ **CXCL5** ]  bind to and activate only CXCR2.  Ref: 9553055 J Biol Chem, 1998
- From these data, it can be concluded that, in addition to IL 8, GCP 2, but not ENA 78  [ **CXCL5** ] , efficiently binds to both CXCR1  [ **IL8RA** ]  and CXCR2.  Ref: 9692902 Eur J Biochem, 1998
- In contrast, only IL 8 and GCP 2 but not ENA 78  [ **CXCL5** ] , competed for 125I IL 8 binding to CXCR1  [ **IL8RA** ] .  Ref: 9692902 Eur J Biochem, 1998

**Non-interactive relationship** (e.g. studied together, co-existance, homology, etc.)

- RESULTS Flow cytometry and radioligand binding data indicate that IL 8, GCP 2, and ENA 78  [ **CXCL5** ]  equivalently reduced CXCR 1  [ **IL8RA** ]  and CXCR  [ **IL8RA** ]  2 cell surface expression by 34% to 54%.  Ref: 10593335 Arch Surg, 1999
- Thus in COPD, in addition to CXCL8 and CXCR1  [ **IL8RA** ] ,  **CXCL5**  and CXCR2 appear to play important roles in the airway neutrophilia characteristic of severe exacerbations.  Ref: 12857718 Am J Respir Crit Care MedAm J Respir Crit Care Med, 1999
- ... GCP 2 was tenfold more potent than ENA 78  [ **CXCL5** ]  on CXCR1  [ **IL8RA** ]  transfectants.  Ref: 9692902 Eur J Biochem, 1998
- ... for ENA 78  [ **CXCL5** ] , threefold higher concentrations were necessary to obtain a calcium response in CXCR1  [ **IL8RA** ]  transfected cells than in CXCR2 transfectants.  Ref: 9692902 Eur J Biochem, 1998
- By comparison with the NSC and S COPD groups, neutrophilia and gene expression for epithelial derived neutrophil attractant 78  **CXCL5** , interleukin 8 CXCL8, CXCR1  [ **IL8RA** ]  and CXCR2 were each up regulated in the E COPD group p&lt.  Ref: 12857718 Am J Respir Crit Care MedAm J Respir Crit Care Med, 1998
- anti ENA 78  [ **CXCL5** ]  antibody and anti CXCR  [ **IL8RA** ]  2 antibody inhibited the chemotaxis.  Ref: 11861308 Blood, 2002
